# Machine learning-driven exploration of therapeutic targets for atrial fibrillation-joint analysis of single-cell and bulk transcriptomes and experimental validation

**DOI:** 10.3389/fcvm.2025.1652467

**Published:** 2025-11-03

**Authors:** Yicheng Wang, Hong-Yi Yang, Zi-Ao Fan, Jian-Cheng Zhang

**Affiliations:** ^1^ Shengli Clinical Medicine College of Fujian Medical University, Fuzhou, Fujian, China; ^2^ Fuzhou University Affiliated Provincial Hospital, Fuzhou, Fujian, China; ^3^ Department of Cardiology, Fujian Provincial Hospital, Fuzhou, Fujian, China

**Keywords:** atrial fibrillation, machine learning, single-cell, bulk transcriptomes, therapeutic targets, network physiology

## Abstract

**Background:**

To explore new therapeutic targets and strategies for atrial fibrillation (AF) by analyzing gene expression profiles of AF patients using machine learning techniques combined with transcriptomic data, and to uncover the potential molecular mechanisms underlying AF.

**Methods:**

Transcriptomic datasets associated with AF were obtained from the GEO database. After batch effect removal and normalization, differential gene expression analysis was performed to identify differentially expressed genes (DEGs). Gene Ontology (GO), Kyoto Encyclopedia of Genes and Genomes (KEGG), and Disease Ontology (DO) enrichment analyses were conducted to explore the functions and pathways of these DEGs. Three machine learning algorithms, Least Absolute Shrinkage and Selection Operator (LASSO), Support Vector Machine—Recursive Feature Elimination (SVM-RFE), and random forest (RF), were applied to screen key genes related to AF. A nomogram model was developed based on the identified key genes, and its diagnostic performance was evaluated. Single-cell transcriptome analysis was performed to investigate the cell-type-specific expression patterns of these key genes. Finally, Real-time PCR (RT-qPCR) and western blot (WB) analyses was performed on right auricular tissue from patients with atrial fibrillation and control samples.

**Results:**

A total of 64 DEGs were identified, including 27 upregulated and 37 downregulated genes. Enrichment analyses revealed that these genes were involved in biological processes such as positive regulation of muscular systemic processes, immune responses, and calcium signaling pathways. Three machine learning algorithms identified six key genes for AF. The nomogram model based on these six genes demonstrated excellent diagnostic performance with an AUC of 0.97. Single-cell transcriptome analysis showed specific expression patterns of these key genes in different cell types. Additionally, immune infiltration analysis indicated changes in the immune microenvironment in AF patients. qPCR and WB analyses also indicated that the differences in mRNA and protein expression levels of these six molecules between the control group and the atrial fibrillation group were consistent with the results of transcriptome analysis.

**Conclusion:**

This study provides new insights into the molecular mechanisms of AF and offers potential non-invasive biomarkers for AF diagnosis. The identified key genes and constructed model may facilitate the development of targeted therapies for AF.

## Introduction

Atrial fibrillation is one of the most common sustained arrhythmias in clinical practice, with its prevalence showing a steady upward trend (1, 2). AF not only significantly impairs patients' quality of life but also markedly increases the risk of severe complications such as stroke and heart failure, imposing a substantial economic burden on patients, families, and society (3).

The pathophysiology of AF involves multiple processes, including cardiac electrophysiological remodeling, structural remodeling, aberrant neural regulation, and inflammatory responses (4, 5). Interactions among ion channel dysfunction in atrial myocytes, alterations in intercellular connexins, progression of myocardial fibrosis, and autonomic nervous system imbalance collectively contribute to the initiation and maintenance of AF (6). However, the understanding of these mechanisms remains incomplete, which limits the development of targeted therapeutic strategies (7).

Current treatment options for AF primarily include pharmacological therapy, catheter ablation, and surgical intervention (8, 9). While pharmacological therapy is effective in controlling ventricular rate and preventing thromboembolism, long-term use is often associated with adverse effects, and some patients exhibit poor responsiveness to medication (10). Catheter ablation, as a curative approach, has limited success rates and carries a risk of recurrence. Surgical treatment, being highly invasive, is applicable only to specific patient populations (11). Overall, existing therapies fail to fully meet the clinical needs of AF patients, highlighting the urgent need to explore novel therapeutic targets and strategies (12).

The rapid advancement of high-throughput omics technologies has enabled comprehensive systemic analysis of biological samples, thereby uncovering disease-related molecular signatures and potential mechanisms (13, 14). Transcriptomics, in particular, plays a critical role in elucidating the relationship between gene expression changes and disease progression, providing a rich resource for cardiovascular research (15, 16).

Continuous progress in machine learning and bioinformatics has provided effective tools for processing and interpreting large-scale omics datasets (13, 17–19). Machine learning algorithms can identify patterns, select key features, and construct predictive models from complex datasets, facilitating the discovery of potential biomarkers and therapeutic targets (20–22).

Furthermore, our study aligns with the emerging framework of Network Physiology, which emphasizes the integration of multi-level biological networks to understand complex physiological systems and disease states. In the context of atrial fibrillation, we explore not only gene-level interactions through protein-protein interaction networks but also cell-type-specific expression patterns and immune microenvironment crosstalk, thereby uncovering the network-based mechanisms underlying AF pathogenesis. The application of machine learning further enables the identification of key network hubs that drive AF progression, highlighting the central role of network analysis in bridging molecular features with clinical phenotypes.

This study aims to systematically analyze the gene expression profiles of AF patients using transcriptomic data and machine learning techniques, with the goal of identifying key genes closely associated with AF pathogenesis and therapeutic responses. Through in-depth investigation of these genes, we aim to uncover the potential molecular mechanisms underlying AF.

## Materials and methods

### Data acquisition

Transcriptomic datasets associated with atrial fibrillation were obtained from the Gene Expression Omnibus (GEO) database. For the discovery phase, we selected three datasets (GSE41177, GSE115574, and GSE79768) based on the following criteria: (1) sample type consisted of human atrial tissue, which is directly relevant to AF pathophysiology; (2) each dataset contained a sufficient number of both AF and control samples to ensure analytical robustness; (3) they were generated using comparable high-throughput platforms (Affymetrix or Illumina) to minimize technical batch effects. Other AF-related datasets in GEO were excluded if they had a small sample size (*n* < 5 per group), were derived from non-cardiac tissues for the discovery analysis, or lacked clear phenotyping. The dataset GSE2240, which is an independent atrial tissue dataset not used in the discovery process, was utilized for external validation of the machine-learning-identified feature genes. Furthermore, the dataset GSE255612, which contains right auricular tissue samples from 18 AF patients and 16 non-AF individuals, was downloaded for subsequent single-cell transcriptomic analysis to explore cell-type-specific expression patterns. The specific distribution of sample sizes for each dataset is shown in Table 1.

**Table 1:** Distribution of sample sizes in each dataset.

Dataset	Platform	Country	Tissue origin	Anatomical location	AF (*n*)	Control (*n*)
GSE41177	GPL570	Taiwan	left atrial appendage	LA free wall	32	6
GSE115574	GPL570	Turkey	left/right atrial appendage	LA/RA free wall	15	15
GSE79768	GPL570	Taiwan	right atrial appendage	LA/RA free wall	13	13
GSE2240	GPL96	Germany	left/right atrial appendage	LA/RA free wall	20	10

### Batch effect removal

Before performing the difference analysis, we merged the three AF datasets (GSE41177, GSE115574, GSE79768). We then corrected for batch effects using the “sva” package of the R language. To assess the effectiveness of this correction, we compared data quality before and after batch removal using principal component analysis (PCA).

### Differential expression analysis

Differential gene expression analysis of the sequencing data was performed using the “limmaa” package in R software to compare samples from the control and experimental groups, thereby identifying DEGs. The criteria for screening DEGs were set as |log2FC| > 0.5 and a *P*-value < 0.05. The results of the differential analysis were visualized using the “ggplot2” package to generate volcano plots and heatmaps. The volcano plot clearly illustrates the distribution of DEGs, including upregulated genes, downregulated genes, and genes with no significant difference in expression.

### GO and KEGG enrichment analysis

GO annotation from the org.Hs.eg.db package (version 3.1.0) in R software was used as the background. Genes were mapped to this background, and GO analysis was subsequently performed using the clusterProfiler package (version 3.14.3). The GO analysis covered three aspects: biological processes (BP), molecular functions (MF), and cellular components (CC), aiming to detect enriched pathways and thereby reveal the cellular functions, signaling pathways, and disease-related differentially expressed gene pathways primarily affected by the candidate target genes. KEGG was used to annotate gene pathways. Enrichment was considered statistically significant when *P* < 0.05.

### DO enrichment analysis

DO enrichment analysis was performed using the org.Hs.eg.db R package (version 3.1.0) to obtain gene annotation information for the gene set. These genes were linked to the DO background dataset to ensure each gene was associated with disease classifications in the DO system. This approach aimed to identify disease processes related to atrial fibrillation treatment responses.

### Machine learning algorithm applications

LASSO regression was employed to identify key genes associated with atrial fibrillation. After preprocessing the candidate differentially expressed genes, LASSO regression was implemented using the glmnet function, treating the data as a binary classification problem. The response variable was extracted from sample names using regular expressions. The model was evaluated by plotting the model object and performing cross-validation via cv.glmnet to determine the optimal lambda value. Finally, genes with non-zero coefficients corresponding to the optimal lambda value were identified as key genes related to the disease status of atrial fibrillation and were output. SVM-RFE analysis was conducted using the “e1071”, “kernlab” and “caret” packages in R. The number of genes corresponding to the minimized cross-validation error in the analysis results was used to determine the count of potential biomarkers identified by SVM-RFE machine learning. Genes with average rankings corresponding to the SVM-RFE analysis were selected as potential biomarkers for AF. Random forest analysis was performed using the “randomForest” package in R. The importance scores of differentially expressed genes were obtained at the point of minimized error on the cross-validation curve. Genes with importance scores exceeding 1 were selected as potential biomarkers for AF. A venn diagram was used to identify the intersection of genes obtained from LASSO, SVM-RFE, and Random Forest analyses. The final set of potential AF biomarkers was derived from the overlapping genes identified by these three machine learning methods.

### Construction of protein-protein interaction (PPI) networks

Protein-protein interaction (PPI) networks were constructed using the GeneMANIA database (http://www.string-db.org/) to explore the regulatory interactions between genes and predict potential regulatory factors. This approach facilitated a deeper understanding of gene relationships and their regulatory mechanisms in the context of atrial fibrillation.

### Development and validation of nomogram

The integrated dataset from GSE41177, GSE115574, and GSE79768 (after batch effect correction) was used as the training set to construct the diagnostic model. To ensure a rigorous evaluation and avoid data leakage, the validation process was strictly separated. In this study, the “rms” package in R software was employed to develop a nomogram model for identifying diagnostic genes in AF. Each candidate gene was assigned a specific score, with the total score being the sum of these individual gene scores. The model's performance was first evaluated internally on the training set. To evaluate the model's accuracy, calibration curves were plotted to assess the consistency between predicted probabilities and actual outcomes. Furthermore, decision curve analysis (DCA) was conducted to evaluate the clinical utility of the model. The diagnostic efficacy of six key genes was assessed through receiver operating characteristic (ROC) curve analysis. Finally, the robustness of the model was validated using the independent external validation set (GSE2240), which was not involved in any prior steps of differential expression analysis or machine learning feature selection.

### Single-cell transcriptome analysis

In the single-cell RNA-sequencing (scRNA-seq) analysis pipeline, data normalization is first carried out via the LogNormalize method to guarantee the comparability of gene expression levels across different cells. Then, the FindVariableFeatures method is employed to select highly variable genes (top 2,000). To further eliminate batch effects, the Harmony algorithm is applied for batch correction, enhancing the comparability of data from different experimental batches. Subsequently, dimensionality reduction is performed using principal component analysis (PCA). For cell clustering, the non-linear dimensionality reduction method of *t*-distributed stochastic neighbor embedding (t-SNE) is utilized for analysis. Cell grouping is conducted using the FindClusters function, and the clustering results are optimized by adjusting different resolution parameters. The entire quality control workflow comprises steps such as normalization, batch correction, and dimensionality reduction to ensure the accuracy and reliability of data analysis. With the thresholds of *P* < 0.05 and log2FC > 0.25, “FindAllMarkers” is used to identify differentially expressed genes in each cluster. Based on the unique marker genes in the study, the expression of these marker genes in different clusters is analyzed to annotate the cells.

### Quantitative Rt-PCR analysis

Total RNA was extracted from cardiac tissue using TRIzol Reagent (Invitrogen, CA, USA), and reverse-transcribed into cDNA via the Novo Protein Reverse Transcription Kit (Suzhou, China). Real-time PCR was performed on a Roche LightCycler® 480 Real-Time PCR Apparatus (Bio-Rad, Basel, Switzerland) to detect the expression of C1orf105, DHRS9, CHGB, PDE8B, CSRP3, FCER1G, and β-actin (as a normalization control). The relative expression levels of these hub genes were calculated using the 2−ΔΔCT method. Statistical analysis was conducted with GraphPad Prism, and t-tests were applied for two groups of data following a normal distribution. A significance level of *P* < 0.05 was adopted. The primer sequences for C1orf105, DHRS9, CHGB, PDE8B, CSRP3, and FCER1G are listed in Table 2.

**Table 2:** The sequences of the primers for qPCR.

Gene symbol	Species	Forward primer	Reverse primer
C1orf105	Human	ATTCACTACAGACTGCCCATTCT	CGTTGTCTTGCCTATTGGTTCC
DHRS9	Human	GGCTTTGGAAACTTGGCAGC	TCGGTCACATCCAGAAGCAC
CHGB	Human	GCCAGATCGGAAACACATGC	CGTCGTTTGTCCACCTCAGA
PDE8B	Human	CAAACTCAGAACTTCGATGCAGA	CTTCATGGTCATCCGATACTCG
CSRP3	Human	GTGCTATGGGCGCAGATATGG	CTCGGACTCTCCAAACTTCGC
FCER1G	Human	CTCCAGCCCAAGATGATTCCA	CTTTCGCACTTGGATCTTCAGTC

### Western blot analysis

Total protein was extracted from right auricular tissues of AF patients and non-AF controls using RIPA lysis buffer containing protease and phosphatase inhibitors. Protein concentrations were determined using a BCA Protein Assay Kit (Beyotime, China). Equal amounts of protein (20 μg per lane) were separated by 10% SDS-PAGE and transferred onto PVDF membranes (MeilunBio, China). After blocking with 5% non-fat milk for 1 h at room temperature, the membranes were incubated overnight at 4°C with primary antibodies against C1orf105 (1:2,000, Abmart, PH13497), DHRS9 (1:2,000, immunoway, YN0639), CHGB (1:2,000, immunoway, YT6192), PDE8B (1:2,000, Proteintech, 30708-1-AP), CSRP3 (1:2,000, immunoway, YN6528), FCER1G (1:2,000, Abmart, TD13263), and β-actin (1:10,000, immunoway, YM8343) as a loading control. After washing, the membranes were incubated with HRP-conjugated secondary antibodies (1:5,000, Proteintech) for 1 h at room temperature. Protein bands were visualized using an ECL detection system (Tanon, China). The grayscale values of protein bands were analyzed using ImageJ software (National Institutes of Health, USA), and the relative expression levels were normalized to β-actin. Statistical analysis and graph generation for WB data were performed using GraphPad Prism software (version 9.5, USA).

### Statistical analysis

All statistical analyses and gene expression data were processed using R (version 4.4.3). When the data were normally distributed, we compared the two groups using an independent two-sample *t*-test. If the data were not normally distributed, we used the Wilcoxon rank-sum test for intergroup comparisons. A *p*-value of less than 0.05 was set as the threshold for statistical significance.

## Results

### Identification of differentially expressed genes

**Figure 1:**
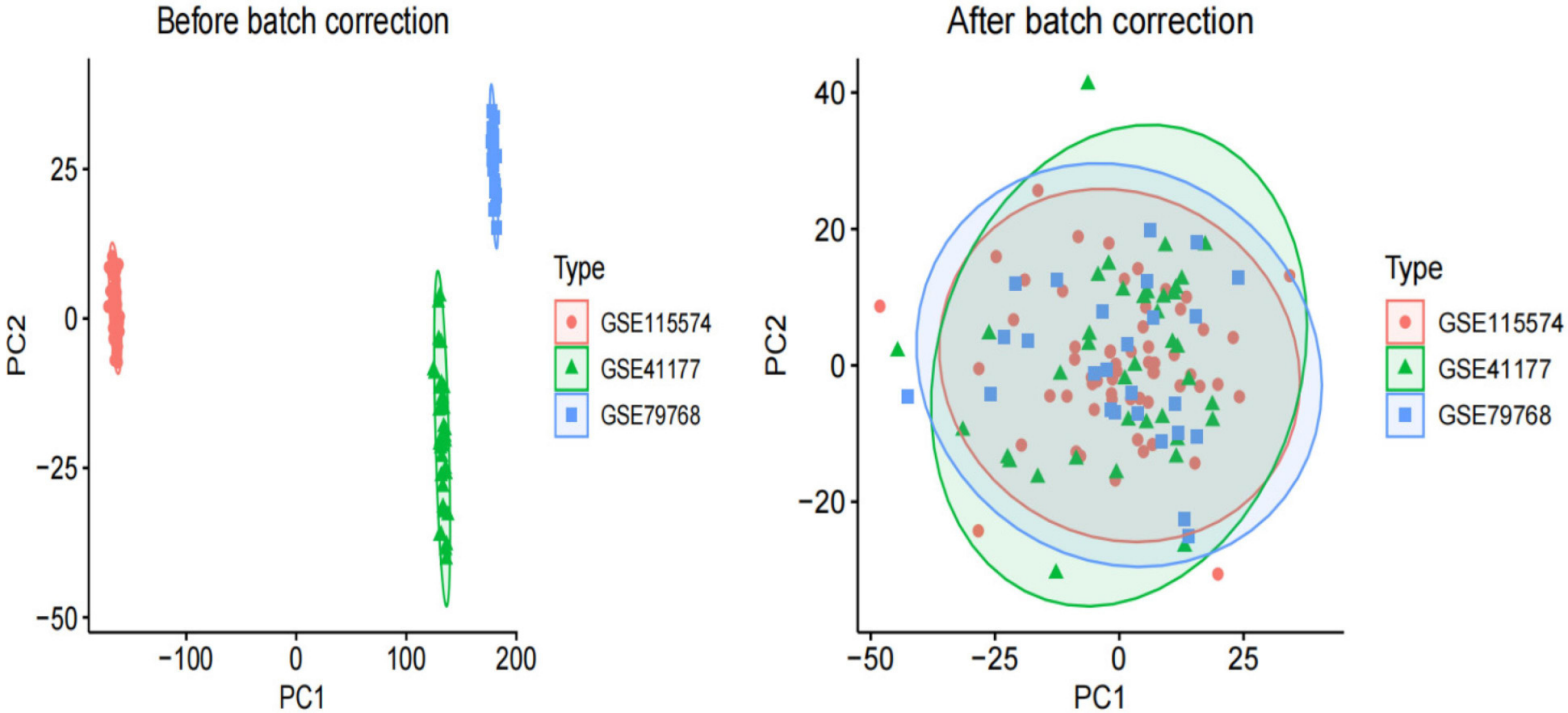
PCA of three original AF datasets prior to batch effect correction and PCA of integrated AF dataset after batch effect correction.

Raw AF and control transcriptome data were obtained from the GEO database, integrated after batch effect removal, and normalized to generate 58 AF cases and 65 control treatment cohorts (Figure 1).

### Identifying of differentially expressed associated with AF

**Figure 2:**
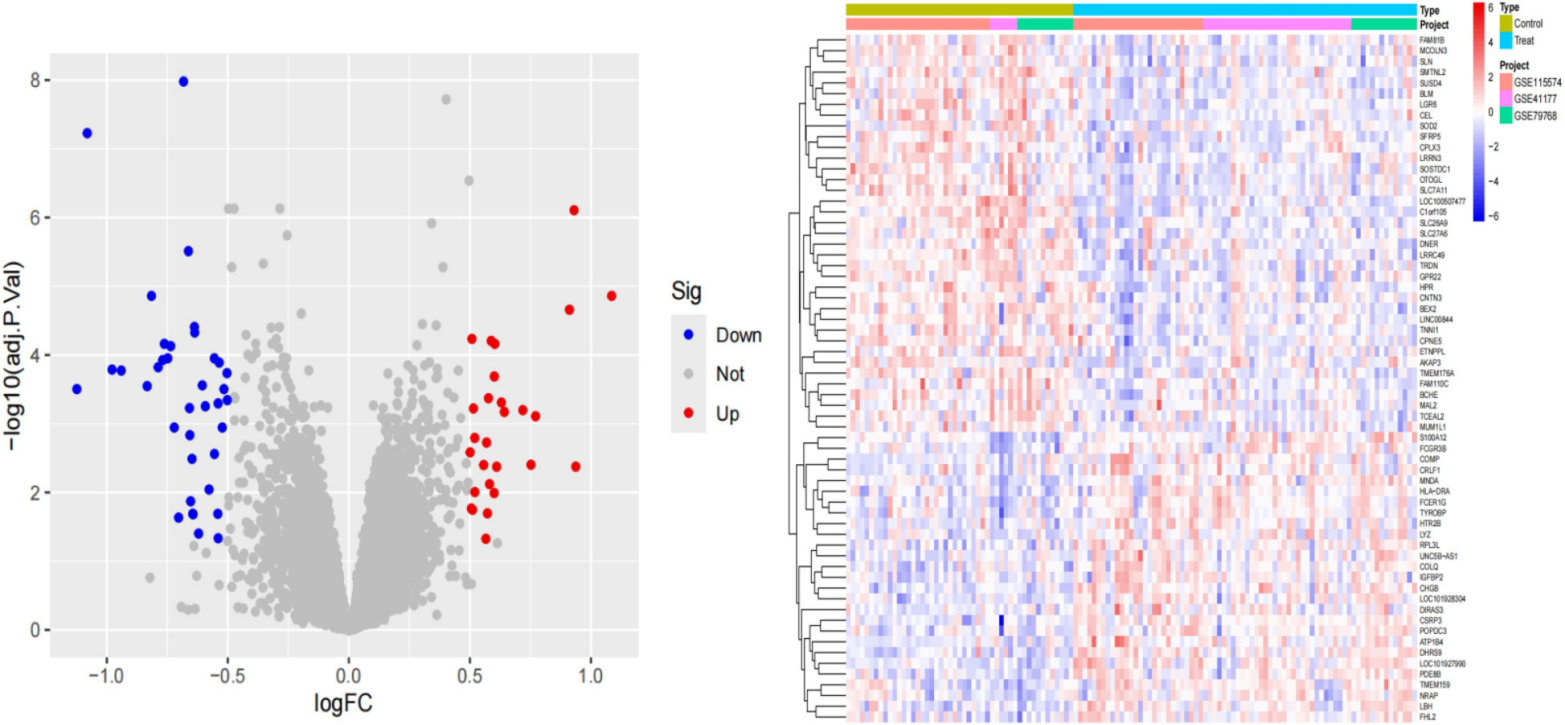
Volcano and Heatmap plots depicting DECs between AF and healthy controls.

We performed differential analysis of the AF cohort to reveal differential genes for AF. A total of 64 deg were identified, of which 27 were upregulated and 37 were downregulated (Figure 2).

### Functional enrichment analysis of AF differential genes

**Figure 3:**
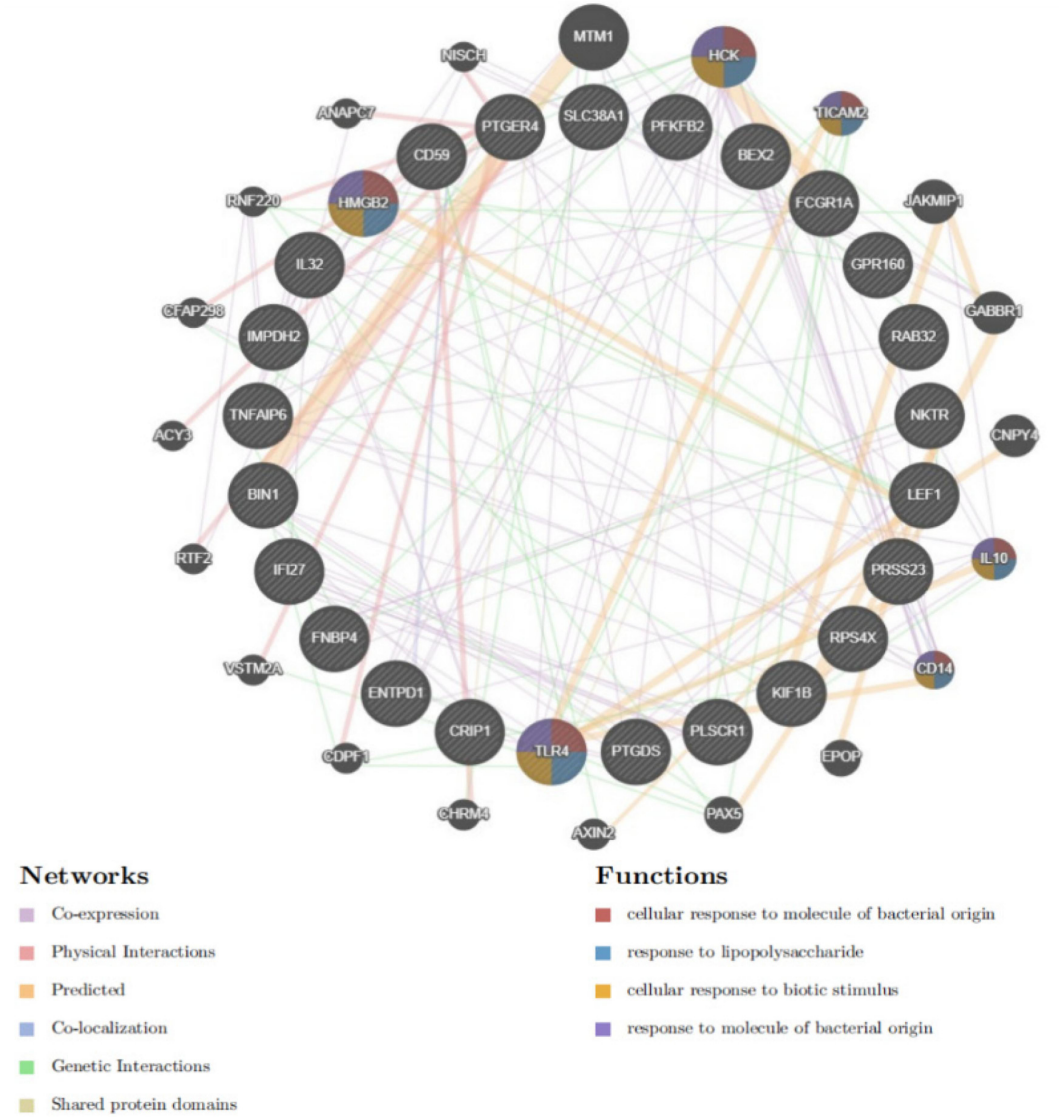
PPI network analysis for differential genes.

Differential gene PPI networks were constructed through the GeneMANIA database (Figure 3) and analyzed for functional enrichment using GO, KEGG, and DO to identify potential mechanisms of action.

**Figure 4:**
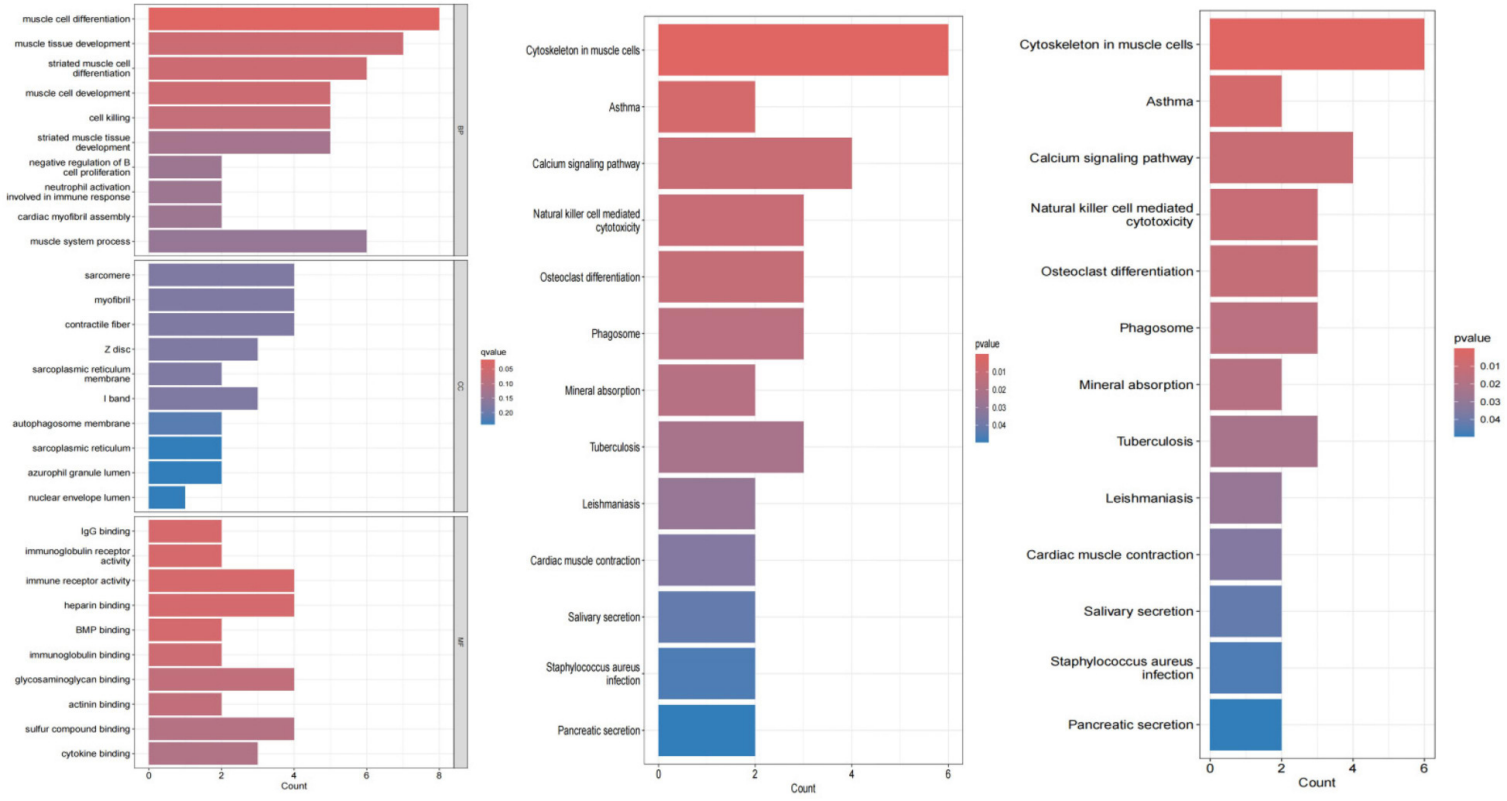
Barplots of GO, KEGG, D0 enrichment analysis results.

The results of the enrichment analysis are shown in Figure 4. In the biological process, AF-related DEGs are enriched in positive regulation of muscular systemic processes. This includes positive regulation of muscle cell development; negative regulation of myocardial fiber assembly; regulation of immune response; positive regulation of muscle tissue development; and negative regulation of muscle cell differentiation. For cellular components, these genes are mainly enriched in cellular structures such as myofibers, sarcoplasmic reticulum, nucleus pulposus lumen, autophagosomal membranes, I-bands, Z-discs, and myogenic fibers. For molecular function, these genes are enriched in a variety of molecular binding activities: cytokine binding; immunoglobulin receptor activity; glycosaminoglycan binding; immunoglobulin binding; BMP binding; heparin binding. These functions are involved in the regulation of the heart and the immune system, suggesting that AF may be closely related to the interaction and signaling of these molecules.

KEGG pathway analysis further revealed significant enrichment of AF-related genes in several biological processes. Specifically, pathways such as pancreatic secretion, salivary secretion, and myocardial contraction, which are closely related to the regulation of cardiac function and the digestive system, showed significant enrichment. At the same time, we also observed enrichment of pathways related to infectious diseases such as Staphylococcus aureus infection and tuberculosis, which may be related to the activation of inflammatory responses in patients with atrial fibrillation. In addition, the enrichment of pathways such as mineral uptake and natural killer cell-mediated cytotoxicity suggests possible immune and metabolic mechanisms involved in AF. The significant enrichment of the calcium signaling pathway is particularly noteworthy because this pathway plays a central role in cardiac electrophysiology and contractile function, and its abnormalities may be directly associated with the development of AF. Finally, the enrichment of cytoskeletal pathways in muscle cells emphasizes the importance of cardiac muscle structure and function in AF.

Disease ontology semantic and enrichment analyses revealed significant associations of AF with multiple biological processes. Specifically, AF was significantly associated with processes such as pancreatic secretion, Staphylococcus aureus infection, salivation, myocardial contraction, leishmaniasis, tuberculosis, mineral uptake, phagolysosomes, osteoclast differentiation, natural killer cell-mediated cytotoxicity, calcium signaling pathways, asthma, and cytoskeleton in muscle cells.

### Analysis of immune cell infiltration in AF

**Figure 5:**
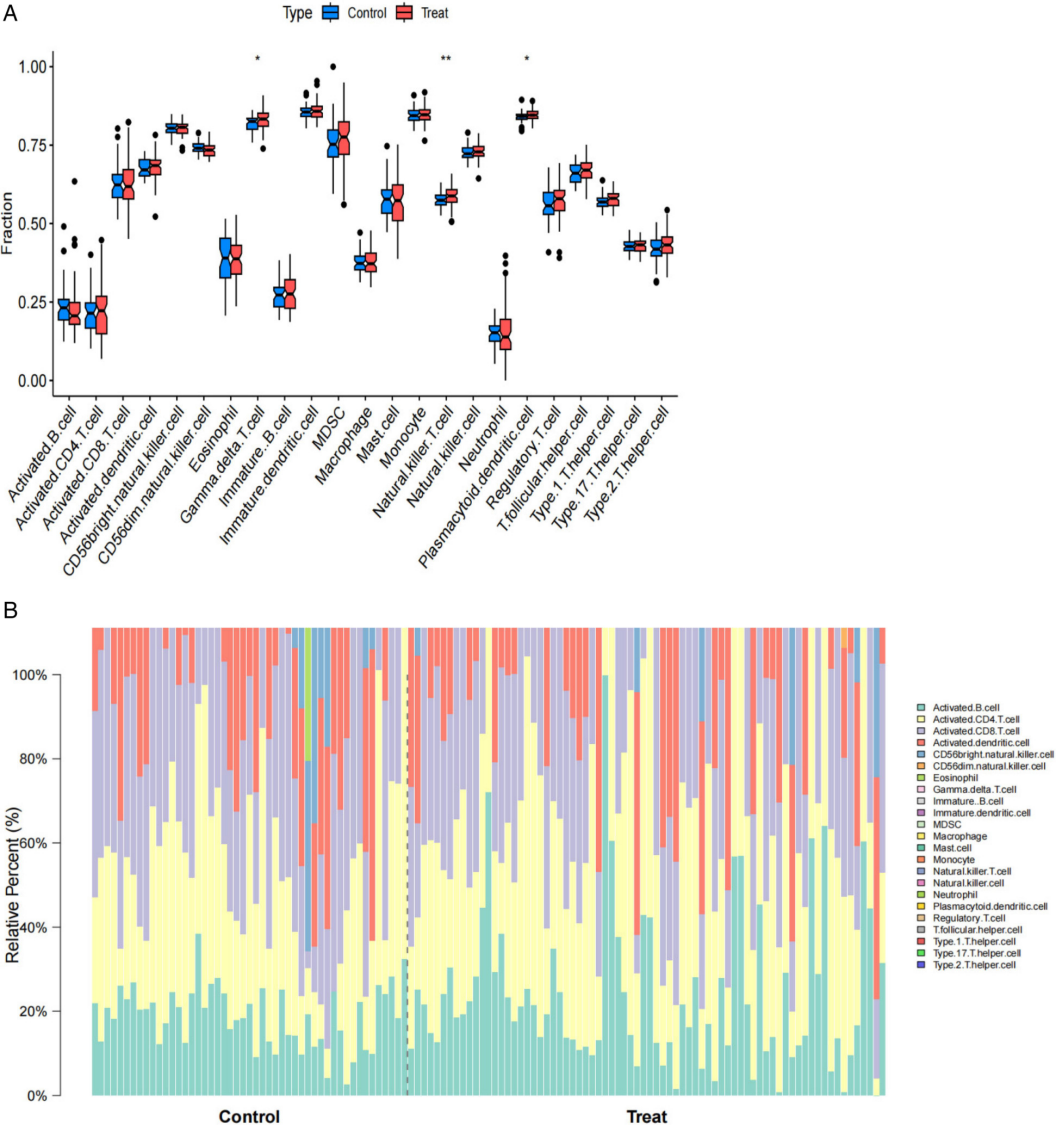
**(A)** Boxplots comparing immune cell abundances between AF vs. controls. **(B)** Barplots comparing imumme cell abundances between AF vs. controls.

Single-sample gene set enrichment analysis (ssGSEA) results for atrial fibrillation revealed functions and pathways associated with immune cell subsets. ssGSEA was used to depict the relative abundance of immune cell subsets in the AF cohort. Samples from the AF cohort showed activated B cells, activated CD4+ T cells, activated CD8+ T cells, activated dendritic cells, CD56bright natural killer cells, CD56dim natural killer cells, eosinophils, γ δ T cells, immature B cells, immature dendritic cells, myeloid-derived suppressor cells (MDSC), as compared to controls, macrophages, mast cells, monocytes, natural killer T cells, natural killer cells, neutrophils, plasmacytoid dendritic cells, regulatory T cells, follicular helper T cells, type 1 helper T cells, type 17 helper T cells, and type 2 helper T cells were enriched. The box line plot further demonstrates that the proportions of macrophages, endothelial cells, and activated dendritic cells were elevated in the atrial fibrillation cohort, whereas the abundance of effector memory CD8+ T cells was reduced compared with the control group. These results suggest changes in the immune microenvironment in the AF cohort, particularly in the composition of specific immune cell subsets (Figures 5A,B).

### Identification of hub genes *via* machine learning

**Figure 6:**
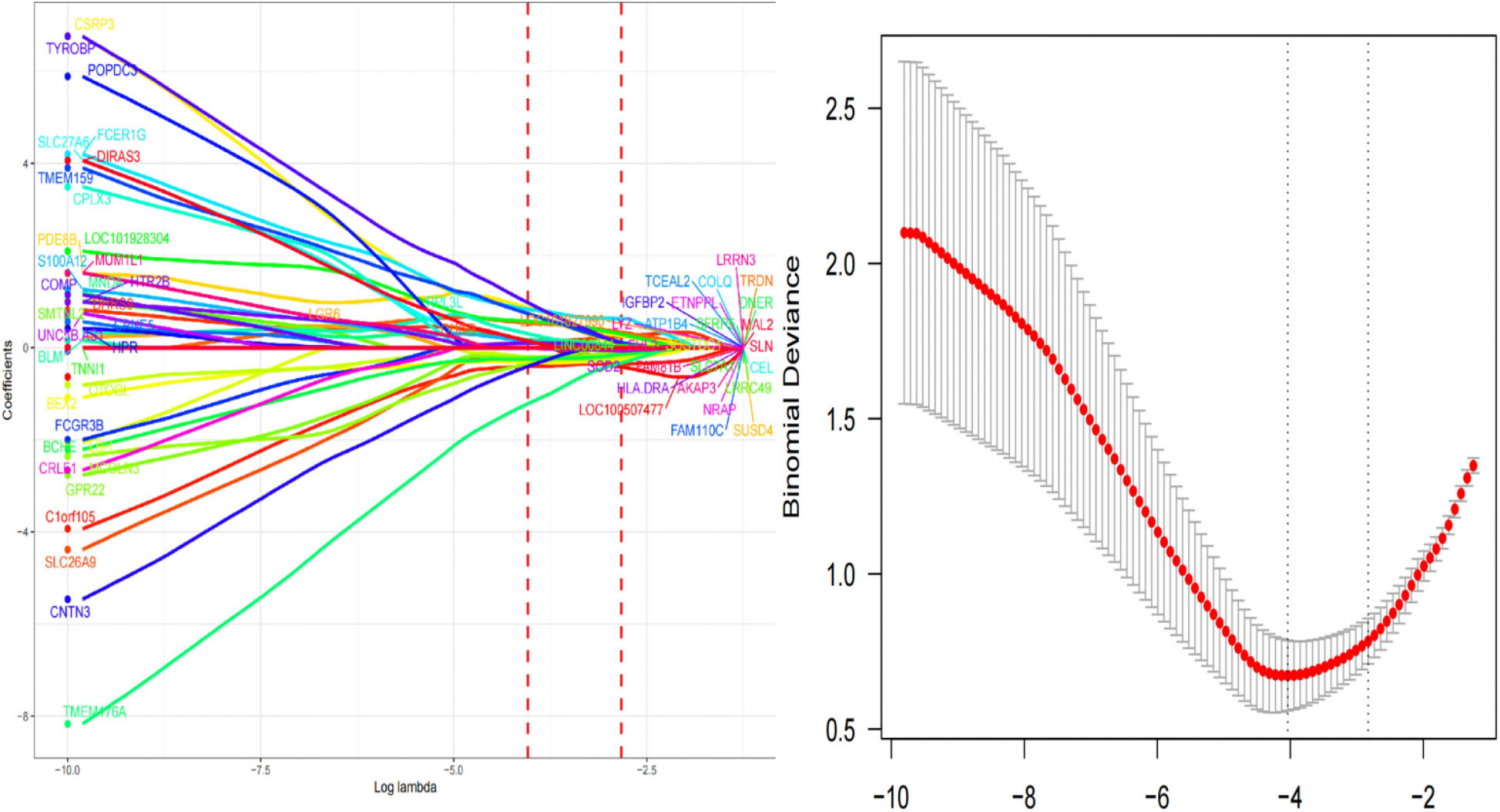
Biomarkers screening and optimal parameter (lambda) in the Lasso model.

**Figure 7:**
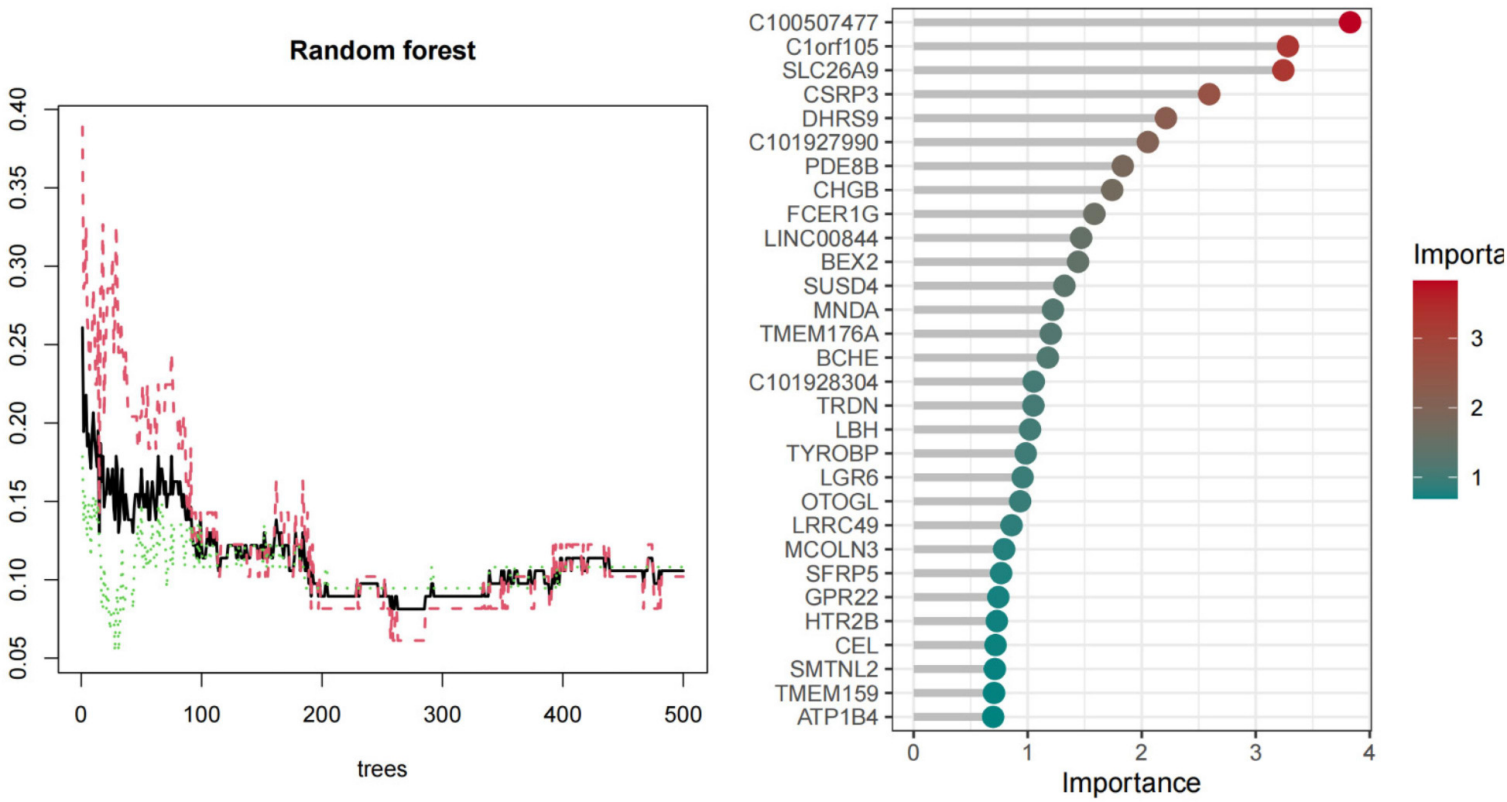
Biomarkers screening relative importance of overlapping candidate top 20 genes calculate& the RF model.

**Figure 8:**
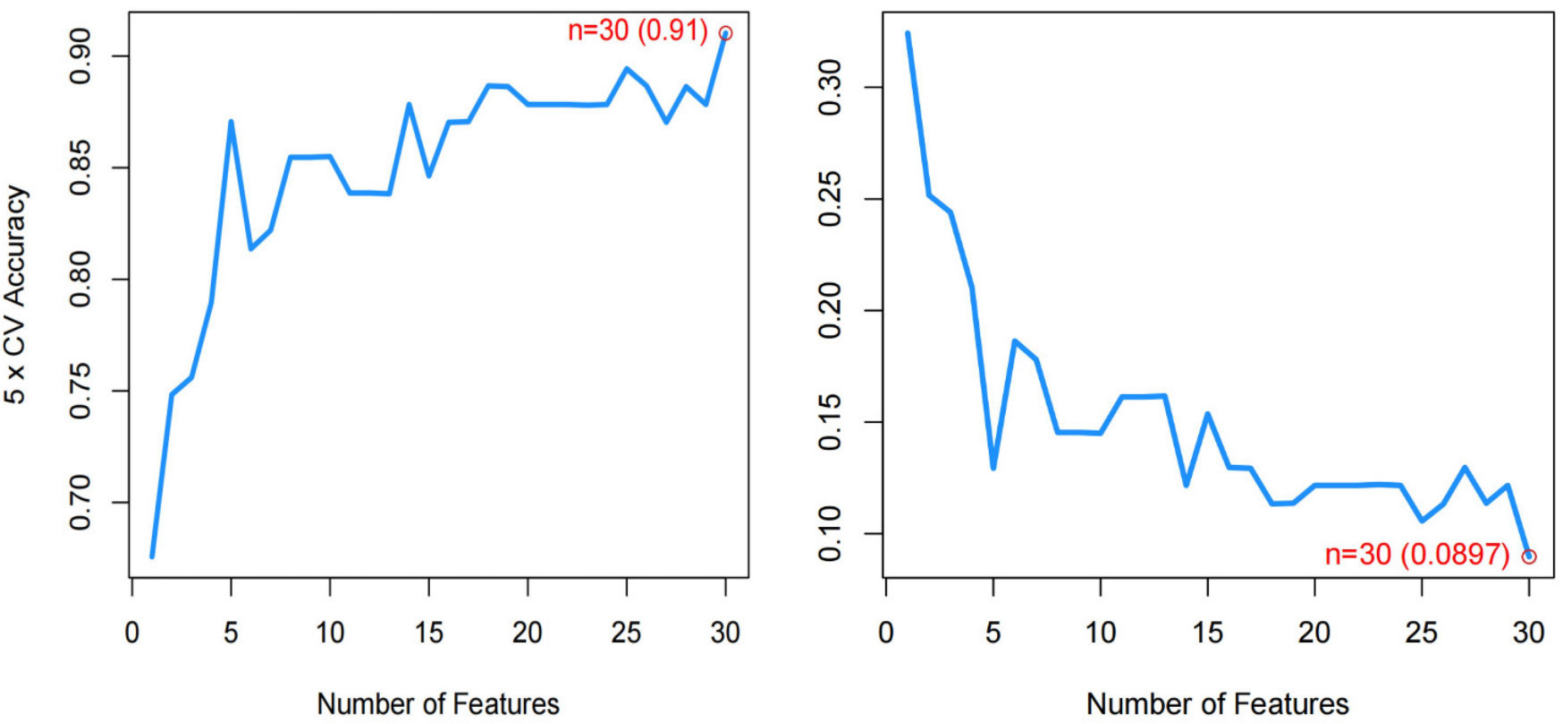
The curve with the highest and lowest biomarker screening accuracy in the SVM-RFE model.

**Figure 9:**
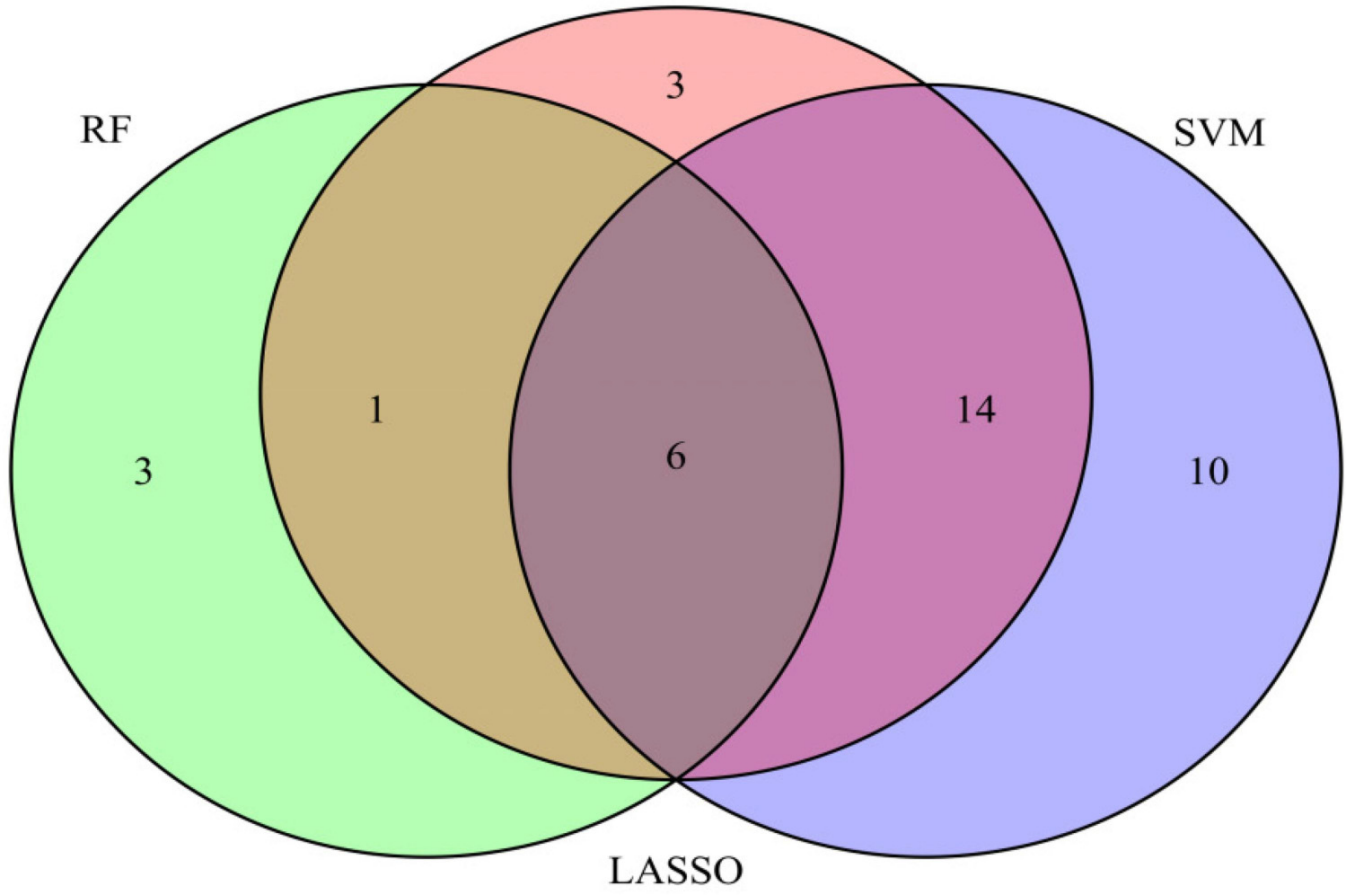
Venn diagram of six candidate genes screened by three machine learning algorithms.

We used three machine learning algorithms, LASSO, RF, and SVM-RFE, to further screen Hub genes for AF. We identified 24 potential candidate biomarkers by the LASSO algorithm (Figure 6). The RF algorithm ranked the genes based on the importance calculation of each gene, and we selected the top 30 as potential candidates for AF (Figure 7). To establish the optimal number of Hub genes, we selected the top 30 genes for the SVM-RFE algorithm results as candidate genes (Figure 8). By intersecting the results of all three algorithms, we identified six Hub genes for AF: C1orf105, DHRS9, CHGB, PDE8B, CSRP3 and FCER1G. The visualization results were shown in Figure 9.

### Diagnostic value assessment

**Figure 10:**
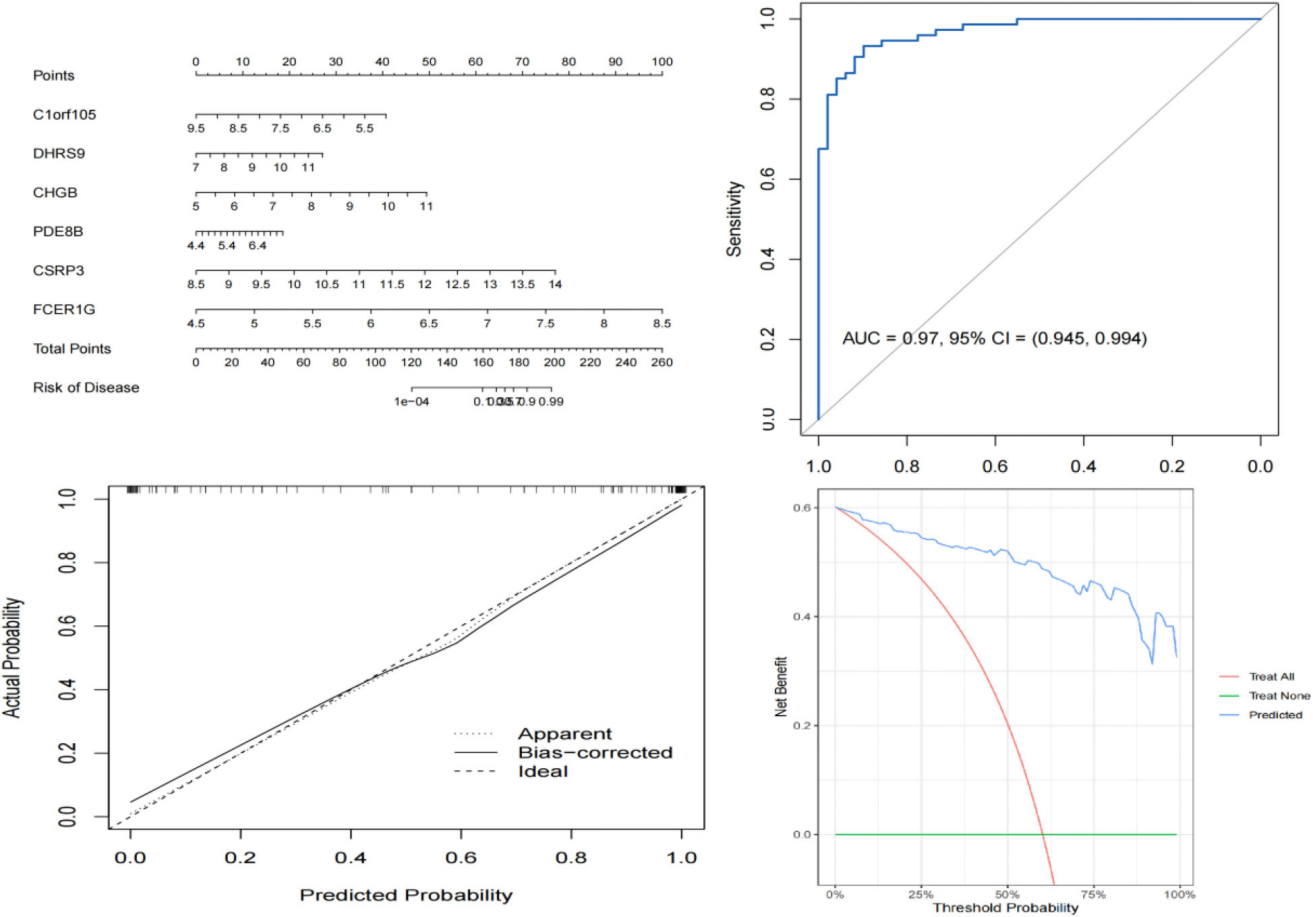
The visible nomogram, ROC curve, calibration curve, DCA curve for diagnosing AF.

**Figure 11:**
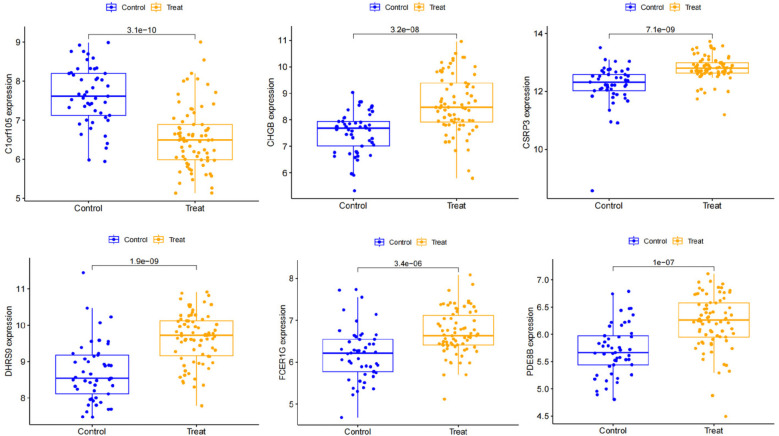
Expression of Hub genes in AF patients compared to normal controls in the training set.

**Figure 12:**
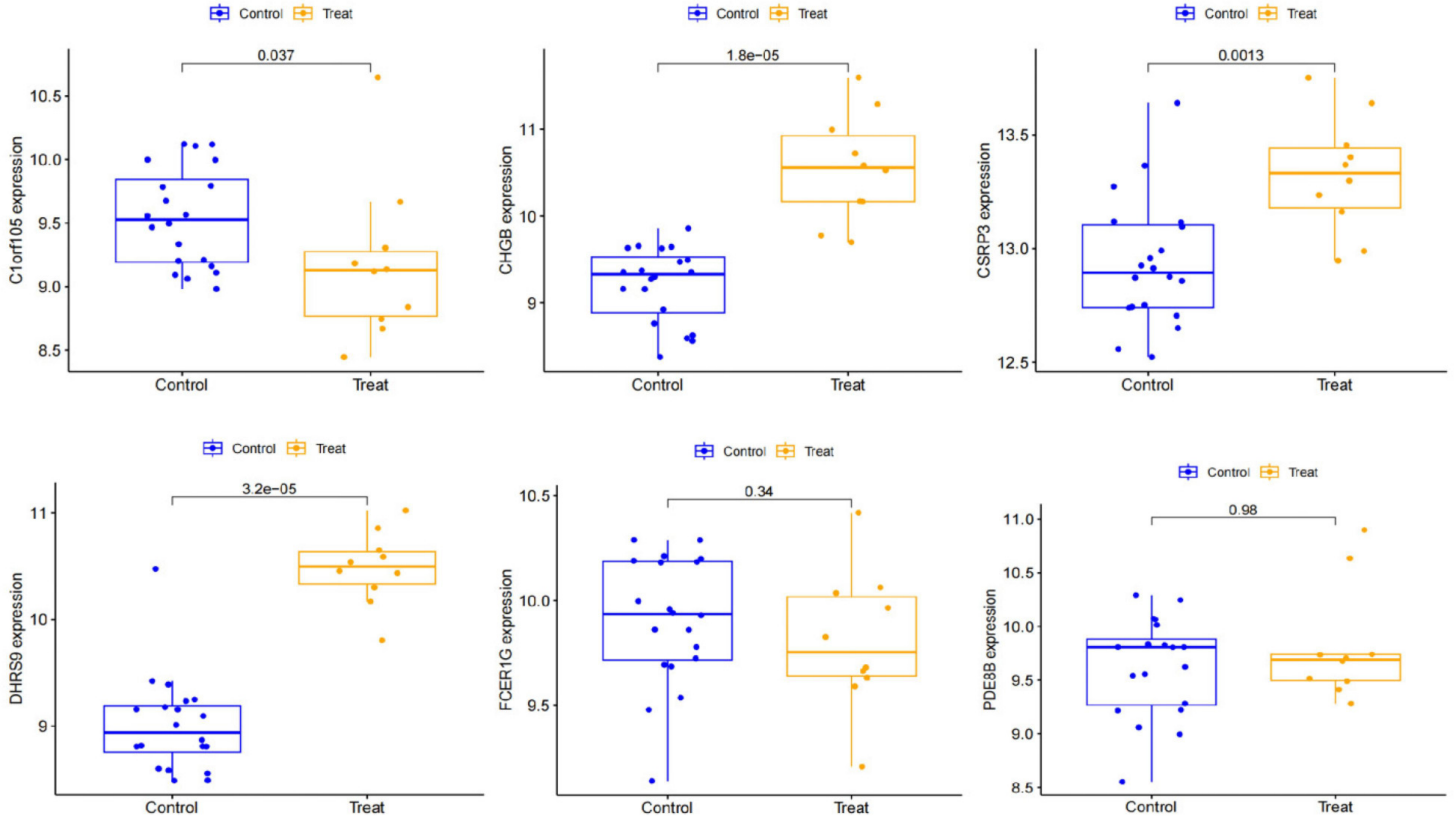
Expression of Hub genes in AF patients compared to normal controls in the validation set.

**Figure 13:**
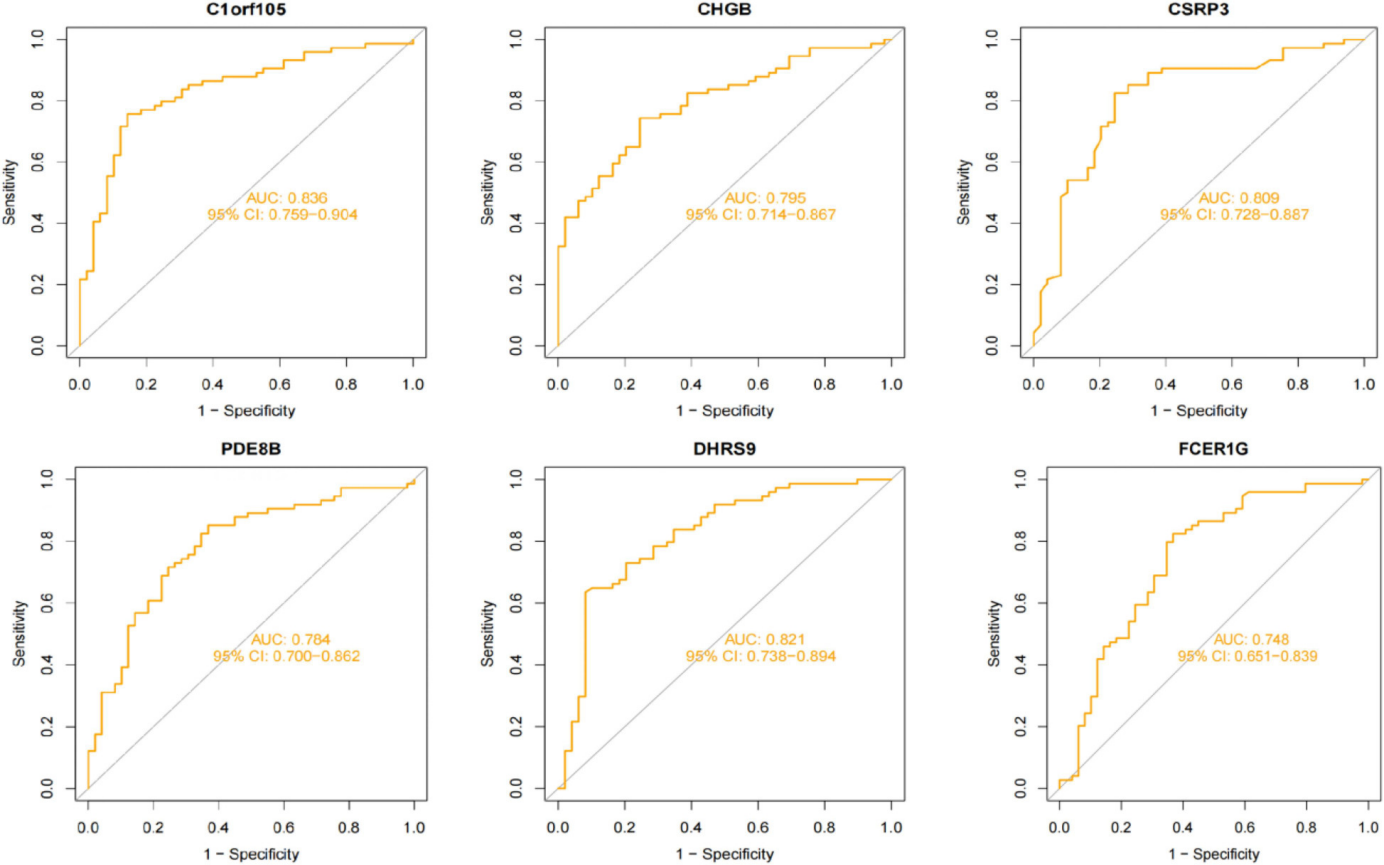
The ROC curve of each candidate genes in the training set.

**Figure 14:**
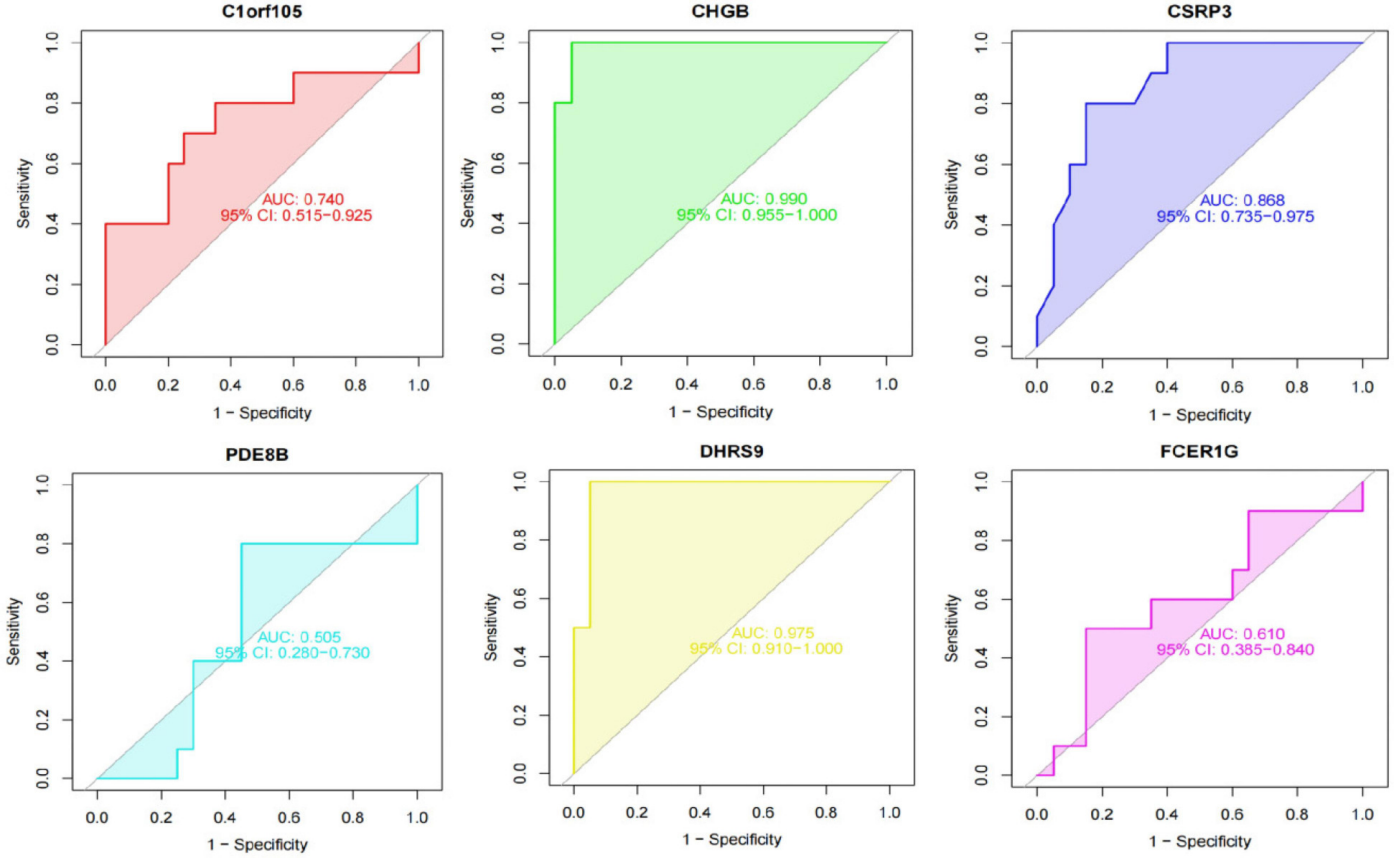
The ROC curve of each candidate genes in the validation set.

We constructed a nomogram model based on the six gene signature. This model demonstrated excellent diagnostic performance, with an AUC of 0.97. Calibration curves validated its accurate predictive capacity for AF. Moreover, DCA results confirmed the clinical applicability of the nomogram model. Collectively, these findings indicate that the nomogram model exhibits robust predictive performance (Figure 10). Additionally, we generated a differential expression box plot of the Hub gene. Finally, we validated the hub genes in GSE2240 by ROC curve analysis. The differential expression results showed that the expression of DHRS9, CHGB, PDE8B, and CSRP3 was up-regulated, and the expression of FCER1G and C1orf105 was down-regulated compared to the control (Figure 11). In the external validation set (Figure 12), the expression of DHRS9, CHGB, PDE8B, CSRP3 and FCER1G was up-regulated, whereas that of C1orf105 was down-regulated. The ROC curve analysis results showed that the AUC of each gene exceeded 0.75, indicating significant diagnostic value. The visualization results are shown in Figure 13. Similarly, in the external validation set (Figure 14), each gene showed great diagnostic value.

### Expression levels in single-cell transcriptome data

**Figure 15:**
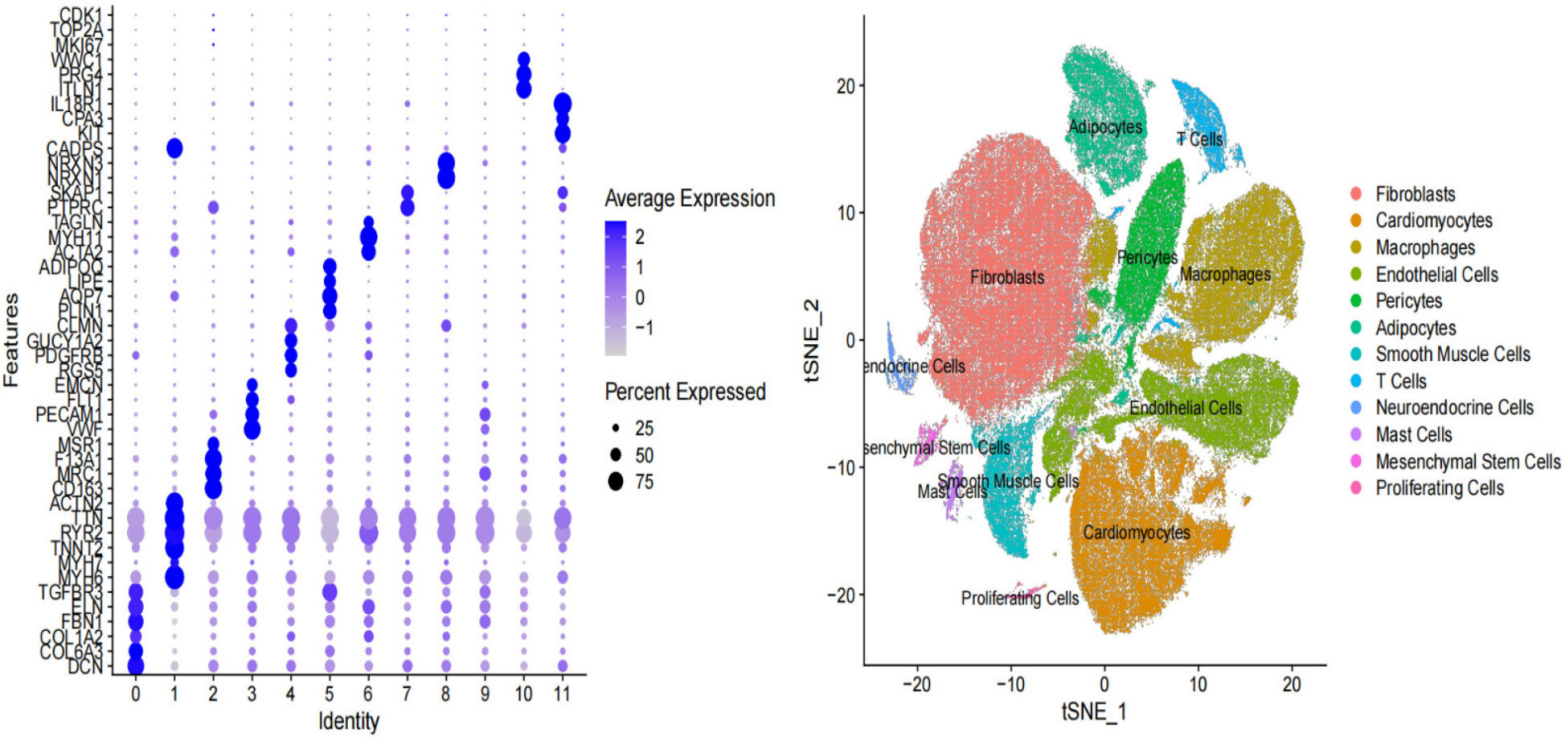
T-SNE clustering visualization for single-cell transcriptome data.

**Figure 16:**
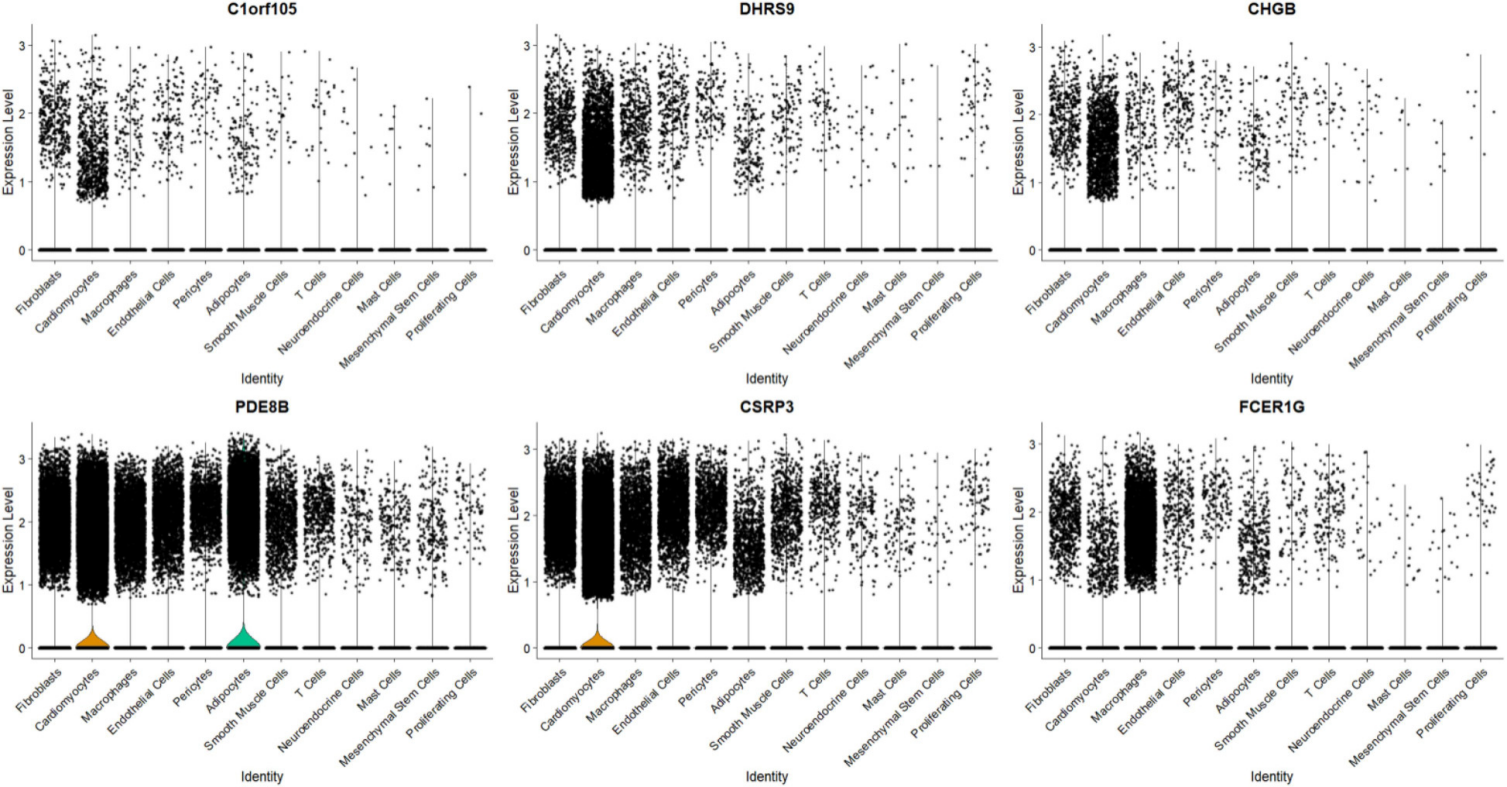
Expression levels of six genes in single-cell treatscriptome data.

**Figure 17:**
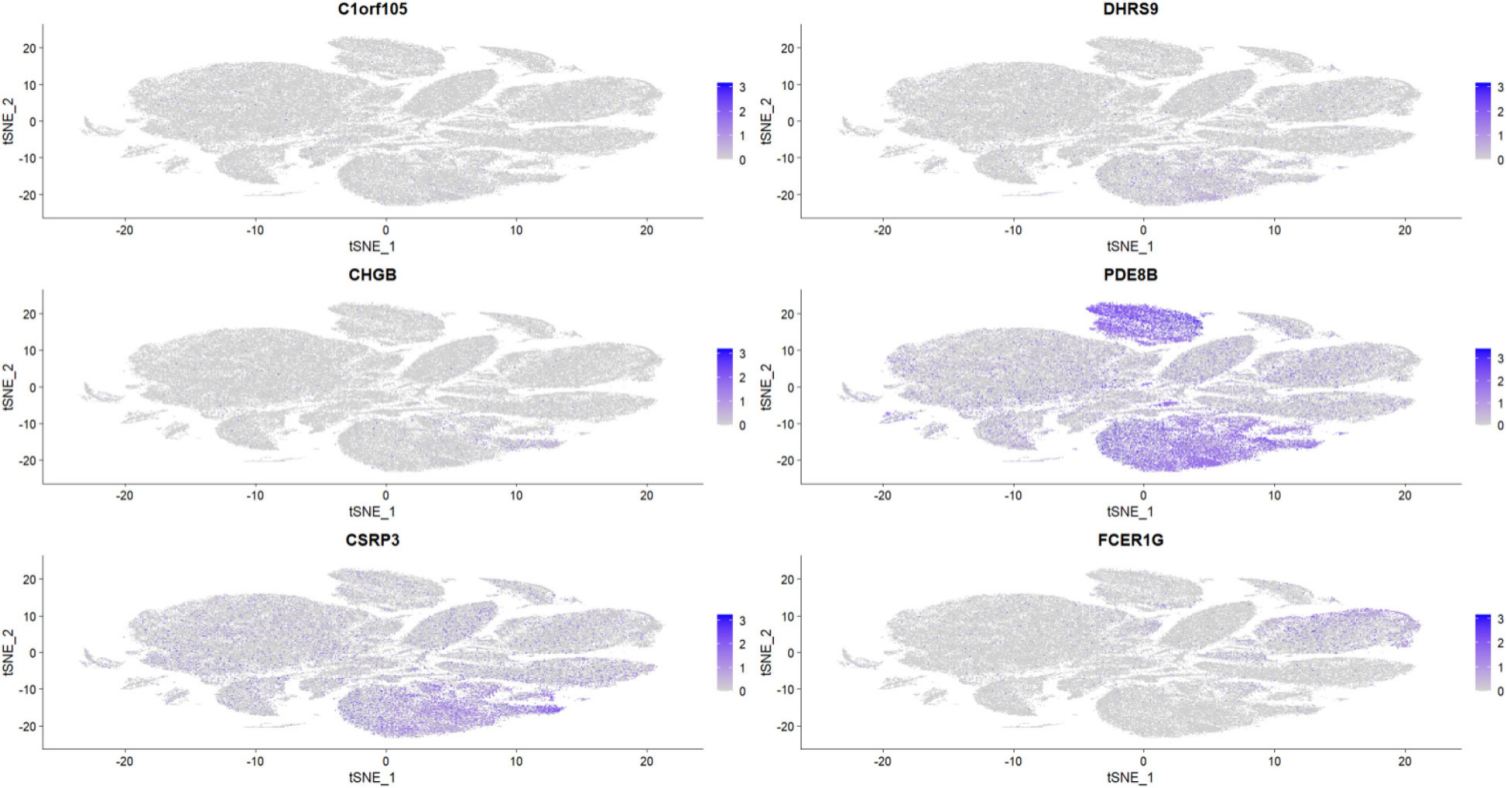
Distribution of six gene expressions in t-SNE space.

To further explore the relationship between the six key genes and atrial fibrillation, we downloaded right auricular tissue samples from 18 AF patients and 16 non-AF individuals in the GSE255612 dataset of the GEO database. After data pre-processing, normalization, scaling, and cell clustering, 12 distinct clusters were identified in the dataset. Upon cell annotation, these clusters were categorized into 12 cell types, namely Fibroblasts, Cardiomyocytes, Macrophages, Endothelial Cells, Pericytes, Adipocytes, Smooth Muscle Cells, T Cells, Neuroendocrine Cells, Mast Cells, Mesenchymal Stem Cells, and Proliferating Cells (Figure 15). Further analysis revealed that DHRS9 and CSRP3 were predominantly expressed in cardiomyocytes, PDE8B in Adipocytes and cardiomyocytes, and FCER1G in macrophages (Figures 16, 17).

To determine if the hub genes were differentially expressed within specific cell types, we performed comparative analysis between AF and control samples for each major cell population, including fibroblasts, cardiomyocytes and macrophages. Violin plots illustrating the expression distribution of the six hub genes in fibroblasts are presented in Supplementary Figures S1, S2. Notably, none of these genes exhibited significant differential expression at the single-cell level within these populations. This indicates that their identification as differentially expressed genes in the bulk tissue analysis is likely attributable to AF-associated changes in the cellular composition of the atrial tissue, such as the expansion of fibroblast and macrophage populations, rather than substantial changes in their expression level within individual cells.

### qRT-PCR experimental validations of the hub genes

**Figure 18:**
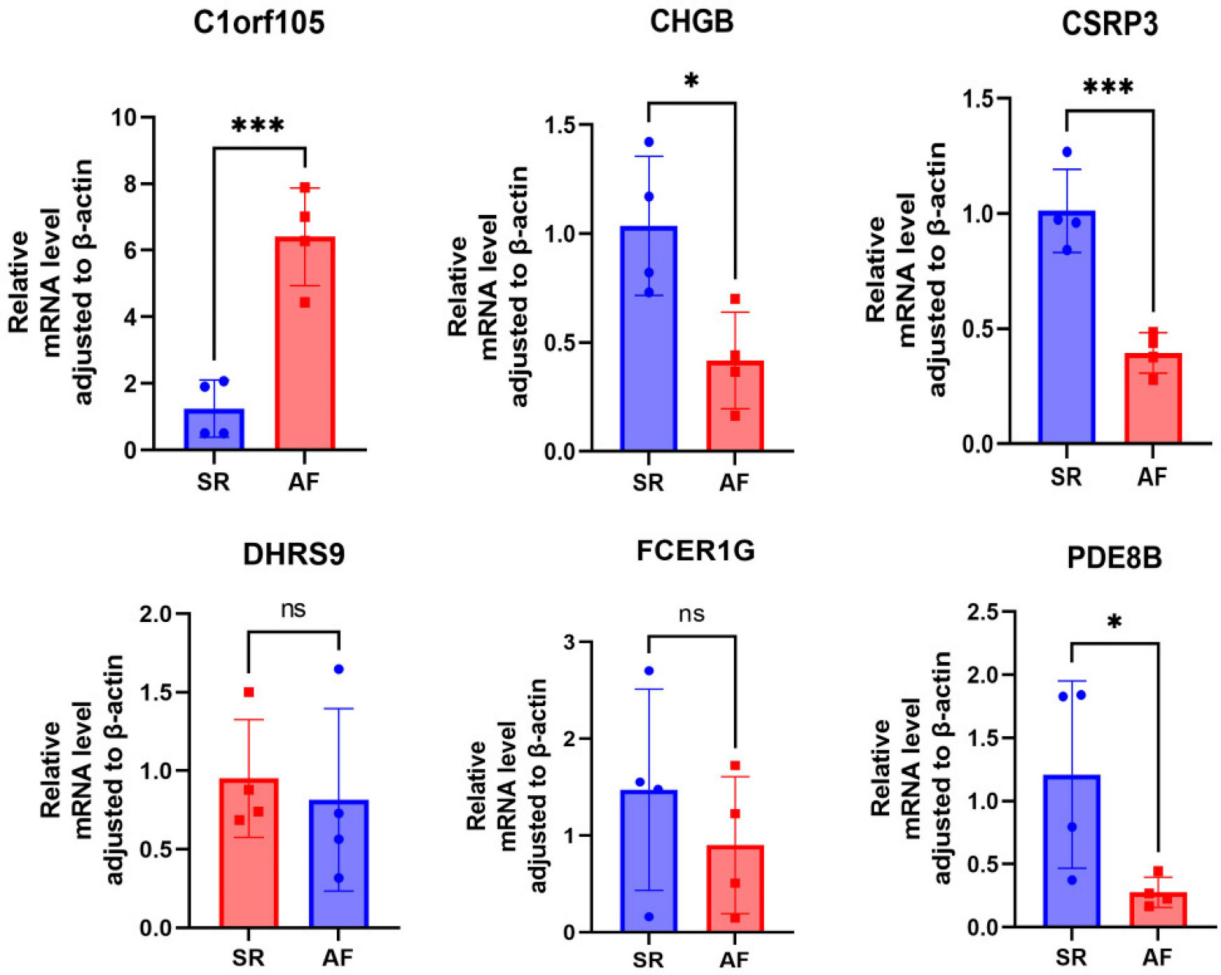
RT-qPCR analysis of six genes expression.

First, we collected right auricular tissues from 4 AF patients and 4 non-AF patients. qRT-PCR results showed that mRNA levels of DHRS9, CHGB, PDE8B, CSRP3, and FCER1G were downregulated in right auricular tissues of patients with AF and upregulated in C1orf105 compared with non-lesional control tissues (Figure 18).

### Western blot experimental validations of the hub genes

**Figure 19:**
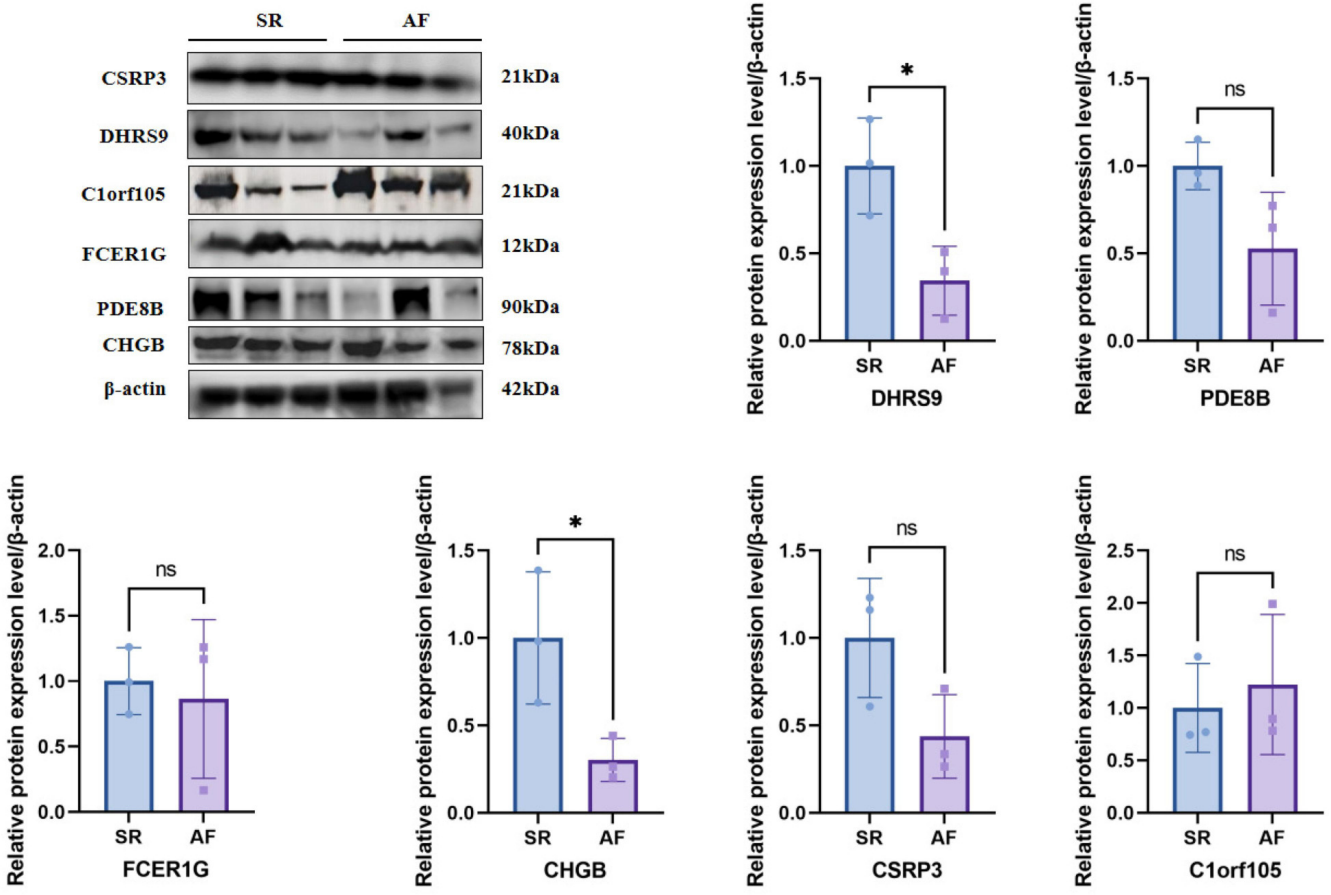
Western blotting and quantitative analysis of six genes expression.

To further validate the protein expression levels of the six hub genes, we performed Western blot analysis on right auricular tissues from 3 AF patients and 3 non-AF controls. Consistent with the mRNA results, the protein levels of DHRS9, CHGB, PDE8B, CSRP3, and FCER1G were significantly downregulated in AF tissues, whereas C1orf105 protein expression was upregulated compared to controls (Figure 19).

## Discussion

This study has systematically revealed the key molecular mechanisms and potential therapeutic targets in the development of atrial fibrillation by integrating single-cell and bulk transcriptomic data with machine learning algorithms.

Our application of machine learning to dissect the molecular underpinnings of AF aligns with a growing trend in cardiovascular medicine, particularly in electrophysiology, to leverage artificial intelligence (AI) for enhanced disease understanding and patient management. For instance, recent advances demonstrate the powerful role of AI and machine learning in electrophysiology, ranging from analyzing electrocardiograms for improved AF detection and classification to predicting ablation outcomes and optimizing patient-specific treatment strategies (23). Our study extends this paradigm by applying similar computational intelligence not to clinical signal data, but to high-dimensional transcriptomic data. This approach allows us to move beyond correlation towards identifying causative molecular features and cell-type-specific expressions that underlie the AF substrate. By integrating bulk and single-cell RNA sequencing with robust machine learning algorithms, we demonstrate how AI-driven bioinformatics can uncover novel, interpretable biomarker signatures that may inform both mechanistic biology and future precision medicine approaches in AF.

Several hub genes closely related to AF have been identified (C1orf105, DHRS9, CHGB, PDE8B, CSRP3, FCER1G). Functional enrichment analysis indicates that calcium signaling pathways, immune microenvironment imbalance, and myocardial structural remodeling play a central role in AF. Single-cell transcriptomic data further reveals the cell—type—specific expression patterns of these hub genes.

DHRS9 is specifically highly expressed in cardiomyocytes, suggesting it may play an important role in cardiomyocyte electrophysiology or structural remodeling (24). DHRS9 encodes a member of the dehydrogenase/reductase family 9 involved in retinoic acid metabolism, and retinoic acid signaling has been proven to be related to cardiac development and fibrosis regulation (25). In this study, the significant differential expression of DHRS9 may reflect myocardial cell metabolic reprogramming in AF patients, leading to abnormal calcium signaling pathways, thereby inducing arrhythmias. In addition, the association of DHRS9 with cardiomyocyte-related pathways, such as myocardial contraction and myofibril assembly, implies that it may be involved in AF progression by regulating the contractility of cardiomyocytes.

CSRP3 is highly expressed in cardiomyocytes, and its encoded protein is involved in sarcomere assembly and cytoskeletal stabilization (26, 27). This study shows that downregulated CSRP3 expression may be closely related to myocardial fibrosis and structural remodeling in AF patients. Previous studies have confirmed that CSRP3 deficiency can lead to the disruption of the Z-disc structure in cardiomyocytes, thereby inducing arrhythmias (28). The significant enrichment of CSRP3 in “myofibril” and “Z-disc” cell components further supports its key role in maintaining the structural integrity of cardiomyocytes. Moreover, the interaction of CSRP3 with calmodulin may indirectly influence the occurrence of AF by regulating calcium ion homeostasis.

The dual-expression pattern of PDE8B in adipocytes and cardiomyocytes reveals the potential role of metabolic regulation in AF. PDE8B encodes phosphodiesterase 8B, which is involved in energy metabolism and signal transduction by degrading cAMP (29). This study finds that abnormal expression of PDE8B may lead to an imbalance in cAMP levels within cardiomyocytes, thereby affecting calcium ion release. The KEGG-enriched “cardiac muscle contraction” pathway supports this finding. Additionally, the high expression of PDE8B in adipocytes may suggest that adipose tissue-derived factors can regulate myocardial electrical activity through a paracrine pathway, offering a new perspective on the metabolic-electrophysiological coupling mechanism of AF.

The specific high expression of FCER1G in macrophages suggests that it is involved in AF progression through immune-inflammatory pathways. FCER1G encodes the high-affinity IgE receptor γ-chain, a key molecule in the activation of mast cells and macrophages (30). This study shows an increase in macrophage infiltration in AF patients. FCER1G may promote the release of pro-inflammatory factors by activating the NF-κB pathway, thereby aggravating atrial fibrosis and electrical remodeling. Its association with “natural killer cell-mediated cytotoxicity” indicates that it may influence the AF microenvironment by regulating immune cell interactions, providing a potential target for targeted immunotherapy.

As newly-discovered AF-associated genes, the specific functions of C1orf105 and CHGB remain to be further elucidated. C1orf105 is widely expressed in single-cell data and may be involved in atrial remodeling by regulating cell proliferation or apoptosis. CHGB is commonly found in neuroendocrine cells, and its upregulated expression may reflect autonomic nervous system dysregulation in AF patients. This is consistent with previous reports that autonomic imbalance can trigger AF (31). Although the functions of these two genes are not yet clear, their association with “neuroendocrine regulation” and “cell proliferation” pathways suggests their potential role in AF, which needs to be verified through functional experiments.

The biomarkers identified in this study have distinct translational pathways depending on their primary source of expression. Tissue-based markers, such as CSRP3 and DHRS9 which are highly expressed in cardiomyocytes, directly reflect the pathophysiological processes of atrial remodeling, fibrosis, and electrophysiological dysfunction. They represent promising therapeutic targets for interfering with the core mechanisms of AF. However, their clinical application as diagnostic tools is limited by the invasiveness of obtaining cardiac tissue. In contrast, the detection of key genes like FCER1G and PDE8B in peripheral blood mononuclear cells (PBMCs), as revealed by our single-cell analysis, offers a promising avenue for non-invasive diagnosis. Blood-based biomarkers could be developed into liquid biopsies for AF screening, risk stratification, and potentially monitoring treatment response. It is important to note that while blood-based markers provide high clinical applicability, their expression levels may reflect systemic states such as inflammation or metabolic alterations, which could be influenced by comorbidities. Therefore, the integration of tissue-specific mechanistic insights with blood-based non-invasive detection methods could facilitate the development of a comprehensive strategy for managing AF.

Immune infiltration analysis shows that the proportion of macrophages and activated dendritic cells is increased in AF patients, while the number of effector memory CD8+ T cells is reduced. This is consistent with the characteristics of the chronic inflammatory state in AF patients. The nomogram model based on the five-gene signature shows excellent diagnostic performance, and its robustness has been validated in an independent dataset. This finding provides a theoretical basis for the development of non-invasive AF biomarker detection. However, the clinical application of the current model still needs further validation in prospective cohorts, and its value in AF subtype stratification or treatment-response prediction needs to be explored.

In addition, the identification of these hub genes and their expression patterns in specific cell types provides novel insights into the pathophysiology of AF. For instance, the high expression of DHRS9 in cardiomyocytes and its association with metabolic reprogramming highlight the importance of metabolic alterations in AF. This could lead to the development of therapeutic strategies targeting metabolic pathways to modulate cardiac electrophysiology and structure. Similarly, the role of CSRP3 in maintaining cardiomyocyte integrity and its link to fibrosis suggest that preserving or restoring its function might mitigate AF progression. Moreover, the dual expression of PDE8B in adipocytes and cardiomyocytes underscores the complex interplay between metabolic tissues and cardiac function, indicating that targeting adipocyte-derived factors could be a novel approach to manage AF.

The immune-related findings, particularly the overexpression of FCER1G in macrophages and the increased infiltration of macrophages in AF patients, emphasize the inflammatory nature of AF. This supports the potential of immunotherapeutic strategies in AF management. The association of FCER1G with immune cell interactions and its role in promoting pro-inflammatory cytokines through the NF-κB pathway offer specific targets for intervention. Modulating the immune response in AF could not only reduce inflammation but also prevent adverse structural remodeling.

Overall, this study bridges the gap between transcriptomic data and functional insights, providing a comprehensive view of AF mechanisms. It highlights the importance of integrating multi-omics data with advanced analytical techniques to uncover disease mechanisms and identifies potential therapeutic targets. Future research should focus on validating these findings in larger, diverse cohorts and exploring the functional roles of these genes through experimental models to translate these insights into clinical applications. Despite the limitations of this study, including its retrospective design and the need for further experimental validation, the identified genes and pathways present promising avenues for developing novel diagnostic tools and personalized treatment strategies for AF.

## Conclusion

This study reveals key molecular mechanisms and potential therapeutic targets for AF. It identifies six genes closely related to AF and demonstrates their specific expression patterns in different cell types. The constructed nomogram model shows excellent diagnostic performance and provides a basis for developing non-invasive biomarkers for AF. However, further experimental validation is needed for clinical application.

## Data Availability

The original contributions presented in the study are included in the article/Supplementary Material, further inquiries can be directed to the corresponding author.
