# Supplementary figures and images for: Machine learning-driven exploration of therapeutic targets for atrial fibrillation-joint analysis of single-cell and bulk transcriptomes and experimental validation
Source: Front Cardiovasc Med. 2025 Nov 3;12:1652467. doi: 10.3389/fcvm.2025.1652467 (PMC12620476; doi:10.3389/fcvm.2025.1652467)

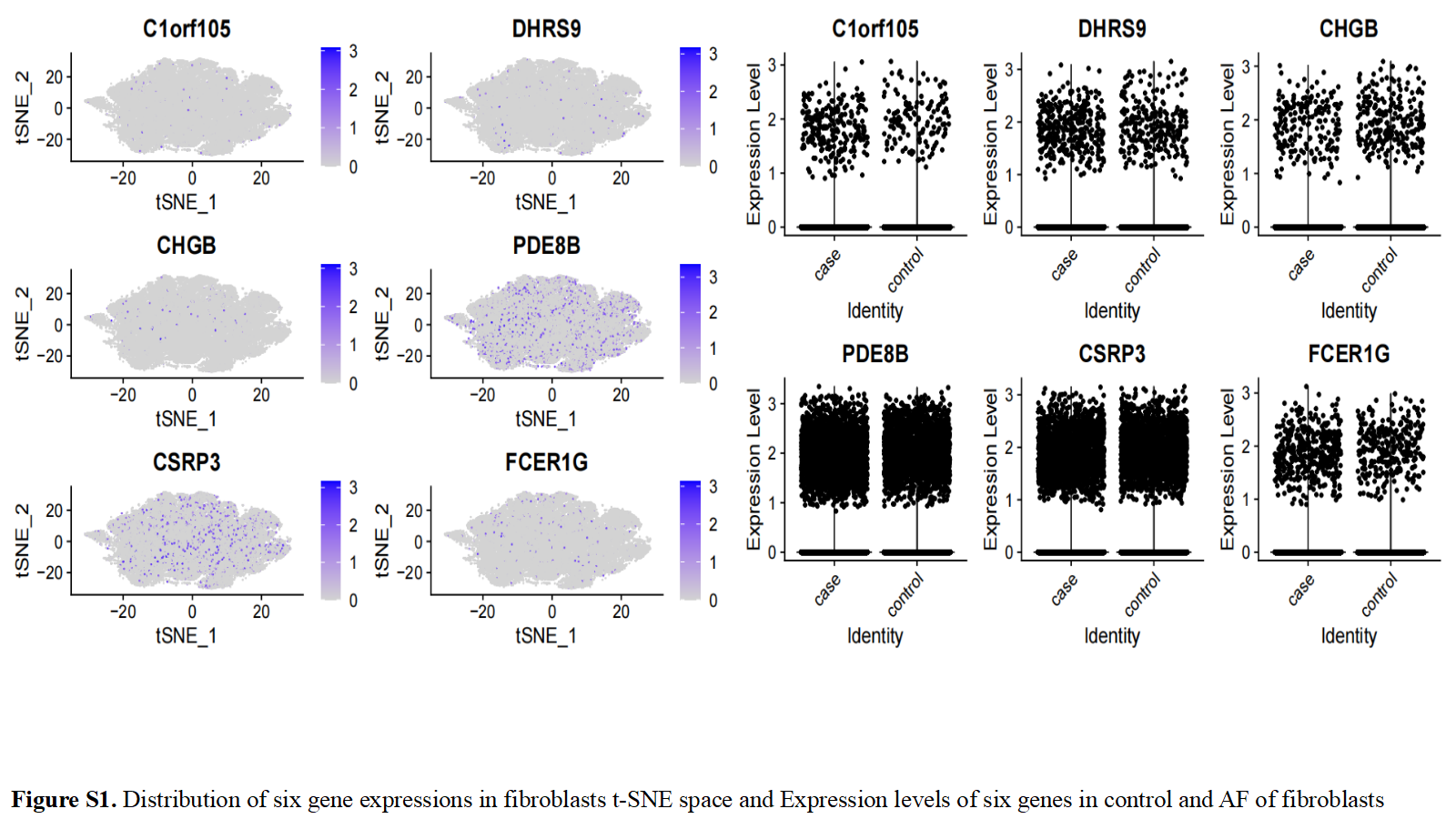

Supplement: Supplementary file 1 [file Image1.tif]

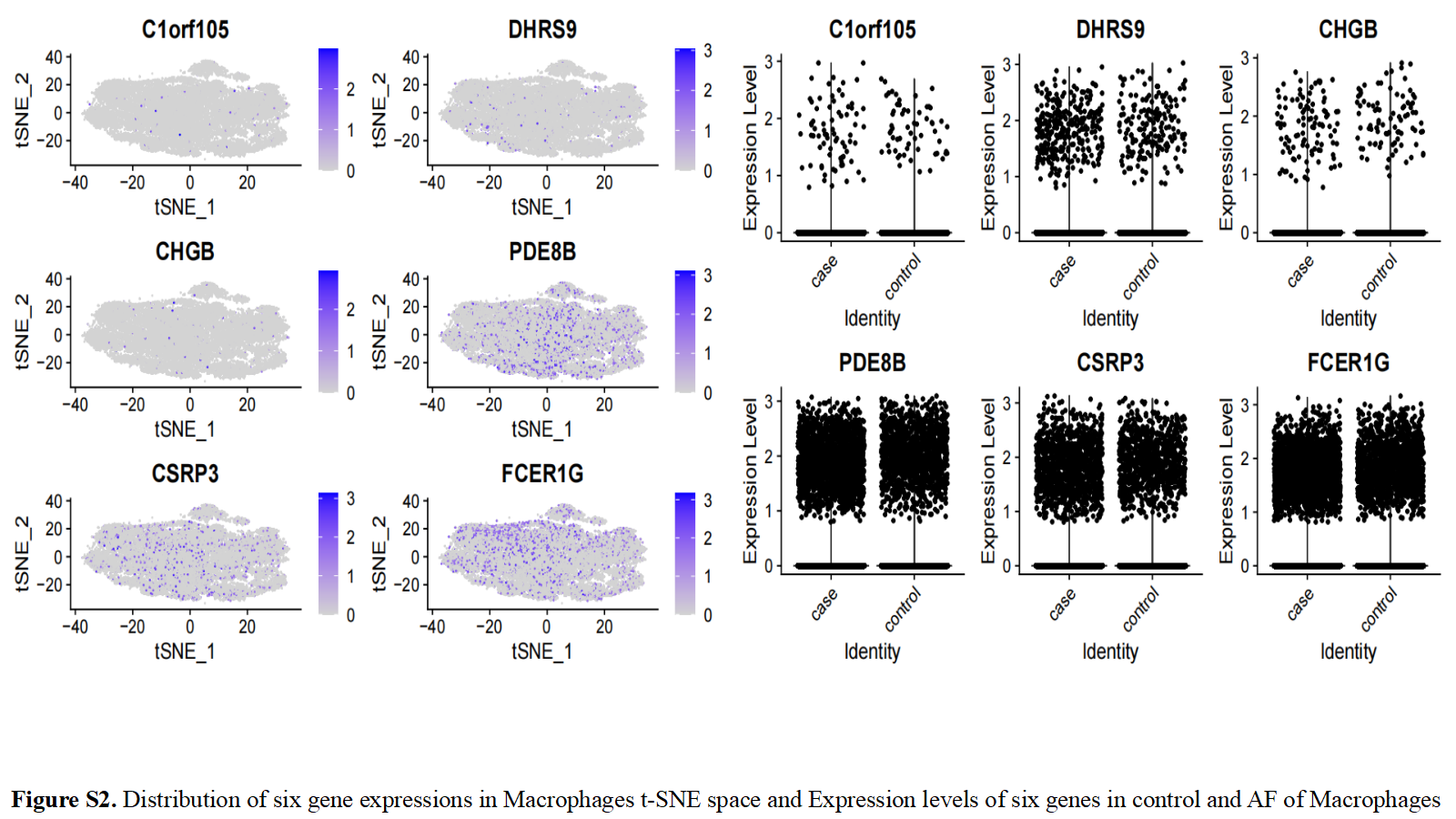

Supplement: Supplementary file 2 [file Image2.tif]
